# The high toxicity of DSB-clusters modelling high-LET-DNA damage derives from inhibition of c-NHEJ and promotion of alt-EJ and SSA despite increases in HR

**DOI:** 10.3389/fcell.2022.1016951

**Published:** 2022-10-03

**Authors:** Veronika Mladenova, Emil Mladenov, Shipra Chaudhary, Martin Stuschke, George Iliakis

**Affiliations:** ^1^ Department of Radiation Therapy, Division of Experimental Radiation Biology, University Hospital Essen, University of Duisburg-Essen, Essen, Germany; ^2^ Institute of Medical Radiation Biology, University Hospital Essen, University of Duisburg-Essen, Essen, Germany; ^3^ Institute for Advanced Biosciences, Inserm U 1209 / CNRS UMR 5309 Joint Research Center, Grenoble Alpes University, Grenoble, France; ^4^ German Cancer Consortium (DKTK), Partner Site University Hospital Essen, Essen, Germany; ^5^ German Cancer Research Center (DKFZ), Heidelberg, Germany

**Keywords:** double-strand break clusters (DSB-clusters), high-LET ionizing radiation, DNA end-processing, SCAs-formation, mFISH

## Abstract

Heavy-ion radiotherapy utilizing high linear energy transfer (high-LET) ionizing radiation (IR) is a promising cancer treatment modality owing to advantageous physical properties of energy deposition and associated toxicity over X-rays. Therapies utilizing high-LET radiation will benefit from a better understanding of the molecular mechanisms underpinning their increased biological efficacy. Towards this goal, we investigate here the biological consequences of well-defined clusters of DNA double-strand breaks (DSBs), a form of DNA damage, which on theoretical counts, has often been considered central to the enhanced toxicity of high-LET IR. We test clonal cell lines harboring in their genomes constructs with appropriately engineered I-SceI recognition sites that convert upon I-*Sce*I expression to individual DSBs, or DSB-clusters comprising known numbers of DSBs with defined DNA-ends. We find that, similarly to high-LET IR, DSB-clusters of increasing complexity, i.e. increasing numbers of DSBs, with compatible or incompatible ends, compromise classical non-homologous end-joining, favor DNA end-resection and promote resection-dependent DSB-processing. Analysis of RAD51 foci shows increased engagement of error-free homologous recombination on DSB-clusters. Multicolor fluorescence *in situ* hybridization analysis shows that complex DSB-clusters markedly increase the incidence of structural chromosomal abnormalities (SCAs). Since RAD51-knockdown further increases SCAs-incidence, we conclude that homologous recombination suppresses SCAs-formation. Strikingly, CtIP-depletion inhibits SCAs-formation, suggesting that it relies on alternative end-joining or single-strand annealing. Indeed, ablation of RAD52 causes a marked reduction in SCAs, as does also inhibition of PARP1. We conclude that increased DSB-cluster formation that accompanies LET-increases, enhances IR-effectiveness by promoting DNA end-resection, which suppresses c-NHEJ and enhances utilization of alt-EJ or SSA. Although increased resection also favors HR, on balance, error-prone processing dominates, causing the generally observed increased toxicity of high-LET radiation. These findings offer new mechanistic insights into high-LET IR-toxicity and have translational potential in the clinical setting that may be harnessed by combining high-LET IR with inhibitors of PARP1 or RAD52.

## Introduction

Radiotherapy is an intricate and essential component of present-day cancer therapy and uses either photons in the form of X-rays, or accelerator-produced charged particles – mainly protons and heavy ions (HI) (Allen et al., 2011; Durante et al., 2017; Durante, 2019). The rationale for using charged-particle therapy comes from their favorable physical properties of energy deposition, as defined by the Bragg peak, as well as by the associated increase in LET that is more pronounced and relevant with HI. Thus, compared to X-rays, charged particles can penetrate deeply into the human body to reach and sterilize tumors *via* increased energy deposition. In addition, the increased LET at the Bragg peak induces complex DNA damage that further increases their efficacy.

The key DNA lesion induced by IR and responsible for the effectiveness of radiotherapy is the DSB (Schipler and Iliakis, 2013; Mavragani et al., 2019). DSBs are severe lesions that threaten genomic integrity. If left unrepaired or incorrectly repaired, DSBs lead to cell death – which is the desired outcome for tumor cells during radiotherapy. The increased biological efficacy of high-LET IR is reflected in a large increase in cell killing that is frequently rationalized as deriving from an increase in the yield of “complex” DSBs – an equivocal term with a wide range of connotations (Hada and Georgakilas, 2008; Sage and Harrison, 2011; Cadet et al., 2012; Schipler and Iliakis, 2013). Notably, the adverse biological consequences of increased DSB-complexity underpin the expected therapeutic benefit and guide current efforts for increased implementation of HI in radiotherapy (Schulz-Ertner et al., 2006; Durante et al., 2017; Durante, 2019). Therapies utilizing high-LET radiation will benefit from a better understanding of the molecular characteristics of the associated “complex” DNA damage and its consequences in the repair pathways guarding the integrity of irradiated genomes.

In mammalian cells, four mechanistically distinct DSB-repair pathways have evolved to mitigate the consequences of DSB induction. Homologous recombination (HR) is the only error-free repair pathway that restores both DNA integrity and sequence at the break site, but its engagement is limited to DNA post-replication stages. Classical non-homologous end-joining (c-NHEJ) rapidly processes DSBs throughout the cell-cycle, but frequently causes mutations (Lieber, 2010; Mladenov et al., 2013; Reid et al., 2015) and SCAs that lead to cell death and cancer (Simsek and Jasin, 2010; Soni et al., 2015).

Alternative end-joining (alt-EJ) is thought to engage when HR or c-NHEJ are inactive or fail – hence often termed backup end-joining (Iliakis et al., 2007; Cho and Greenberg, 2015; Mateos-Gomez et al., 2015; Soni et al., 2015; Sallmyr and Tomkinson, 2018; Wang et al., 2020). Alt-EJ operates with slower kinetics and lower efficiency than c-NHEJ, and is error-prone causing deletions and other modifications at the junction, more than c-NHEJ. Alt-EJ also can join unrelated DNA-ends and is therefore considered a dominant source of SCAs (Zhang et al., 2010; Soni et al., 2015; Mladenova et al., 2022). Single-strand annealing (SSA) is also error-prone owing to the large deletions it generates between the homologous DNA segments it requires for normal function, and can be promiscuous in partner selection and form SCAs (Bhargava et al., 2016; Iliakis et al., 2019).

HR, alt-EJ and SSA are classified as DNA end-resection resection (henceforth simply resection) -dependent DSB-repair pathways, because they share this initial processing step (Ceccaldi et al., 2016). During this step, the MRN/CtIP complex orchestrates short-range resection that is followed by BLM-DNA2/EXO1-mediated long-range resection (Sartori et al., 2007; Mladenova et al., 2022). The resulting ssDNA is a prerequisite for the engagement of HR and SSA, and benefits also alt-EJ (Paiano et al., 2021) by exposing microhomologies (4-6bp). In alt-EJ, POL θ facilitates the annealing of resected 3′-tails, and extends one 3′ DNA-end using the annealing partner as a template (Wyatt et al., 2016) to facilitate the ultimate ligation by either Lig1 or Lig3. Notably, excessive resection can have deleterious consequences, including large deletions and SCAs that promote genomic instability and cell death (Bunting and Nussenzweig, 2013).

The divergent properties and fidelities of the four DSB-repair pathways indicate that they cannot be considered as equivalent alternatives of DSB-repair, and suggest that they may actually also serve to accommodate necessities generated from different sources, including the increased complexity of high-LET IR-induced DSBs (Schipler and Iliakis, 2013; Iliakis et al., 2019). Indeed, DSBs induced by high(er)-LET IR have different processing requirements than those induced by low-LET IR, including increased engagement of HR (Zafar et al., 2010; Shibata et al., 2011; Yajima et al., 2013; Grosse et al., 2014; Fontana et al., 2015; Rall et al., 2015). Also, a subset of DSBs induced by high-LET IR cannot be processed by c-NHEJ, and as a consequence c-NHEJ-deficient cells are equally sensitive to high and low-LET IR. Thus, the most consequential effect of high-LET IR and the type of DSBs it induces, is to shunt them from c-NHEJ to resection-dependent processing by HR, alt-EJ and SSA (Wu et al., 2008; Ceccaldi et al., 2016). In line with this, CtIP depletion, which fully suppresses resection, sensitizes cells to high-LET IR and decreases SCAs-formation (Zhang and Jasin, 2011; Davies et al., 2015; Himmels and Sartori, 2016; Ceppi et al., 2020).

However, increased engagement of an error-free DSB-repair pathway in the form of HR is difficult to reconcile with the dramatic increases in radiosensitivity and SCAs-formation that accompany increases in LET (Lee et al., 2011; Durante et al., 2013; Loucas et al., 2013; Soni et al., 2015). Therefore, we hypothesized that after high-LET IR, the associated suppression of c-NHEJ, causes a general increase in resection-dependent DSB-processing. Although this includes increased utilization of HR, it is also associated with increased utilization of alt-EJ and SSA, and we considered plausible that these altered DSB-processing dynamics ultimately tilt the balance towards error-prone processing, causing the radiosensitization observed. Here, we describe experiments designed to test this hypothesis.

Among the levels of DSB-complexity that have been considered hitherto (Schipler and Iliakis, 2013), DSB-clusters may represent the most consequential form, as they destabilize chromatin and interfere with many forms of DSB-repair (Iliakis et al., 2019). There is evidence that the probability of DSB-cluster formation increases with increasing LET of IR (Schipler and Iliakis, 2013; Schipler et al., 2016). In the present study we employ a previously described model system (Schipler et al., 2016) to test the balance between HR, alt-EJ and SSA in the processing of complex DSBs defined as DSB-clusters. The model system allows the induction in rodent cells of multiple single DSBs (simple form), or clusters of two or four DSBs (increasing complexity) with compatible or incompatible ends (another parameter of complexity) and to compare their consequences on DSB-repair pathway engagement.

We show that increased DSB-clustering suppresses c-NHEJ, promotes CtIP-dependent resection and favors HR, alt-EJ and SSA. However, our results show that on balance, despite the relative increase in the utilization of HR, DSB-clusters cause increased cell killing and increased SCAs-formation through SSA and alt-EJ. The similarities in the processing characteristics between high-LET-induced “complex” DSBs throughout the genome, and of well-defined complex DSBs in form of DSB-clusters, at fixed location in the genome, support the hypothesis that DSB-clusters underpin the biological effects of high-LET IR (Friedland et al., 2011).

## Materials and methods

### Cell culture and inhibitor treatment

**FIGURE 1:**
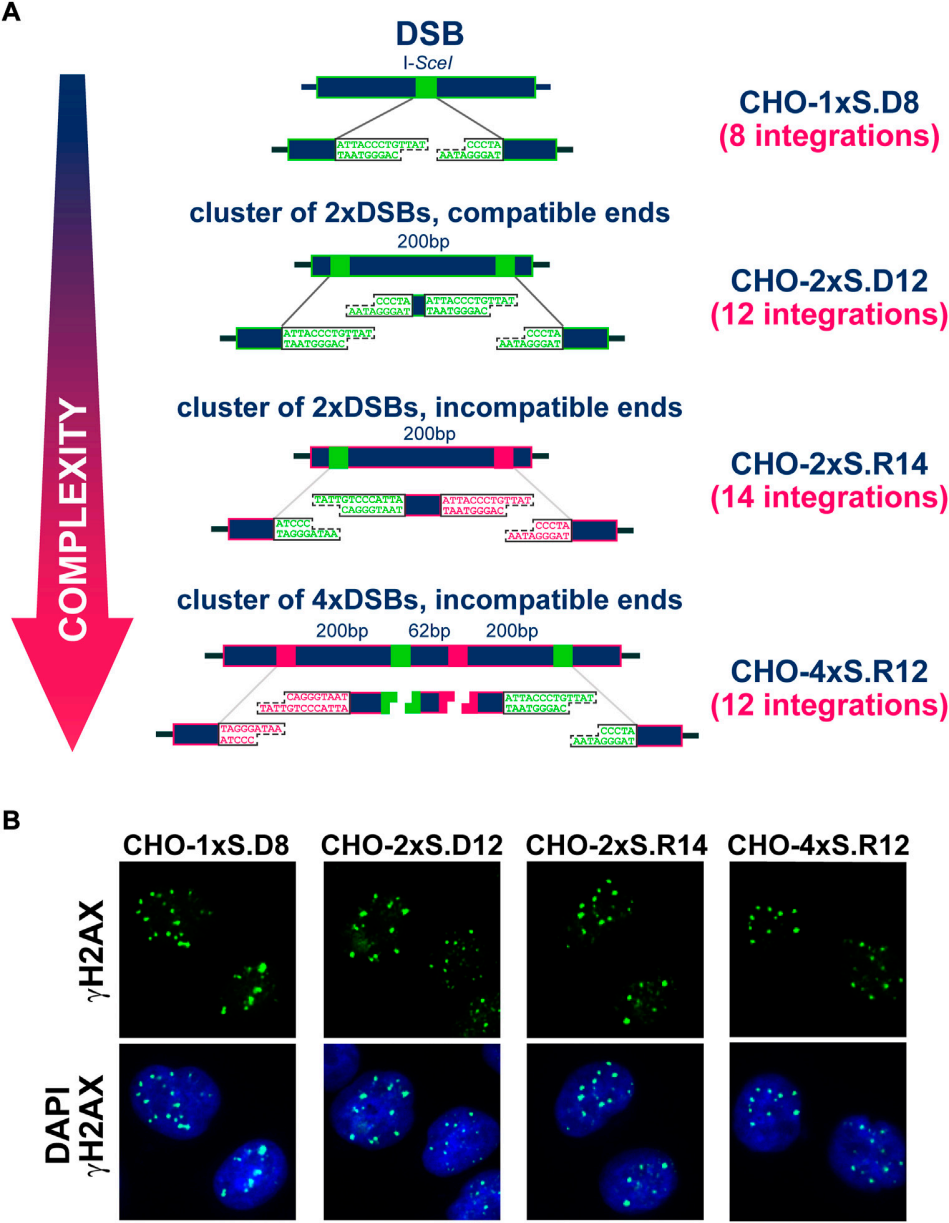
**(A)** Stably integrated constructs carrying different combinations of I-SceI recognition sites engineered at different distances and orientations to model individual DSBs or DSB-clusters. The names of the corresponding clonal cell lines are indicated in dark blue. **(B)** Representative images showing the formation of γH2AX foci in CHO clones transfected with I-SceI expressing plasmid.

Chinese hamster ovary (CHO) clones (Figure 1) carrying stable integrations of I-SceI recognition sequences were generated and described previously (Schipler et al., 2016). The parental cell line CHO10B4 was utilized as a control. Cells were maintained in McCoy’s 5A growth medium (Sigma-Aldrich), supplemented with 5% fetal bovine serum (FBS) (Sigma-Aldrich), at 37°C, in an atmosphere of 5% CO_2_ in air in the presence of 400 μg/ml G418 (Capricorn Scientific). Inhibitors were administered either immediately after transfection or 4 h later. 6-hydroxy-DL-DOPA (RAD52i) (Sigma-Aldrich) was used at a concentration of 20 μM. DNA-PKcs inhibitor NU7441 (DNA-PKcsi) (Tocris Bioscience) was applied at a concentration of 5 μM.

### Colony formation assay

To assess the colony forming ability after expression of I-SceI, 200–1000 cells were plated in triplicate following transfection with pCMV-3xNLS-*ISceI* plasmid. In some experiments, as indicated, the expression plasmid pI*Sce*I-T2A-Trex2-IRES-BFP was utilized (Certo et al., 2012). Cells were grown for 9 days and stained with 1% crystal violet dissolved in 70% ethanol; the dishes were scanned and colonies scored automatically.

### Immunofluorescence (IF) staining and cell-cycle dependent evaluation of DSB-repair foci by quantitative image-based cytometry (QIBC)

For indirect IF analysis, 0.1–0.5 × 10^6^ transfected cells were plated in 12-well plates. For foci scoring in specific phases of the cell cycle, cells were labeled for 30 min with 2 μM EdU just before the indicated times post-transfection for I-SceI expression. EdU negative (EdU^−^) and EdU positive (EdU^+^) cells were analyzed in distinct cell cycle compartments after staining following standard protocols (Mladenov et al., 2020). Cell cycle dependent QIBC evaluation of γH2AX and RAD51 foci was performed as previously described (Mladenov et al., 2020). The following primary antibodies were used: anti-γH2AX (3F2) mouse monoclonal (Abcam), anti-RAD51 (14B4) mouse monoclonal (GeneTex). The secondary antibodies were goat anti-mouse AlexaFluor488, goat anti-mouse AlexaFluor647 (Thermo Scientific).

### SDS-PAGE and western-blot analysis

Protein extracts were prepared and run on SDS-PAGE using standard protocols. Proteins were transferred onto nitrocellulose membranes, which were incubated in Intercept^™^ (TBS) blocking buffer (LI-COR) for 1 h at room temperature, followed by overnight incubation with primary antibody (diluted in Intercept^™^ T20 (TBS) antibody diluent) at 4°C. Membranes were washed three times with TBS-T (0.1% Tween-20, 150 mM NaCl, 25 mM Tris–HCl, pH 7.6) and incubated for 1 h with secondary antibody. The following primary antibodies were used: anti-CtIP (D-4) mouse monoclonal (Santa Cruz Biotechnology), anti-RAD51 rabbit polyclonal (Merck Millipore) and anti-GAPDH mouse monoclonal antibody (Merck Millipore). The secondary antibodies were: goat anti-mouse IgG conjugated with IRDye680 or goat anti-rabbit IgG conjugated IRDye800 (LI-COR Biosciences). The proteins on the membranes were visualized by scanning using Odyssey infrared imaging scanner (LI-COR Biosciences).

### Multicolor fluorescence *in situ* hybridization (mFISH) and classical cytogenetic analysis

To analyze SCAs-formation, mFISH analysis and classical cytogenetic analysis were employed. Briefly, 2.5 × 10^6^ cells transfected with pCMV-3xNLS-*I*Sce*I* plasmid were split in three dishes and plated for 24, 30 and 48 h, respectively. To accumulate cells at metaphase, colcemid (Biochrom AG) was added for 2–3 h at a concentration of 0.1 μg/ml. Metaphase spreads were prepared using standard procedures. mFISH was performed using 12XCHamster Multicolor FISH Probe for Chinese Hamster Chromosomes (MetaSystems Probes) according to manufacturer’s protocol. An automated imaging system (MetaSystems) was used to obtain high quality images of metaphase chromosomes, as previously described (Soni et al., 2019). For analysis, at least 100 metaphases were scored in each of three independent experiments. The number of the SCAs formed in the non-transfected clones is subtracted from the SCAs number in cells transfected with I-SceI expressing plasmid.

Classical cytogenetic methods were also employed, as previously described (Schipler et al., 2016). High quality images of metaphase chromosomes were captured using Zeiss AxioScan.Z1 imaging platform at a magnification of ×40 dry objective. Images were analyzed using the integrated ZEN software. For analysis, at least 100 metaphases were scored in each of three independent experiments.

### Protein knockdown by siRNA interference

For depletion of CtIP, RAD52 and RAD51, a pool of three siRNAs, specific for each protein, as previously described (Kostyrko et al., 2017) (Eurogentec), was introduced by electroporation (Nucleofector^™^ II device, Amaxa Biosystems) following manufacturer’s protocol and program U-23. Briefly, the following oligonucleotides were used for depletion of CtIP: 5′-GUG​CAA​GGU​UUA​CAA​AUA​A-3′; 5′-CAA​AGU​CCC​UGC​CAA​ACA​A-3′; 5′-AGA​AUA​CUC​UCC​AGG​AAG​A-3′ (Eurogentec). Similarly, for downregulation of RAD52, again a cocktail of three specific RNAs was utilized, following the same transfection procedure: 5′-UGA​GAU​GUU​UGG​UUA​CAA​U-3′; 5′-ACU​GCA​UUC​UGG​ACA​AAG​A-3′; 5′-CCC​UGA​AGA​CAA​CCU​UGA​A-3′. For efficient RAD51 ablation the following three specific siRNA sequences were used: 5′-GUG​CCA​AUG​AUG​UGA​AGA​A-3′; 5′-GGG​AAU​UAG​UGA​AGC​CAA​A-3′; 5′-GGC​GUU​CAG​AAA​UCA​UAC​A-3′. The following negative control RNA (ncRNA) sequence was used: 5′-UUCUCCGAACGUGUCACGUdTdT-3′. ncRNA is used for mock-transfection.

### Statistical analysis

The statistical analysis was carried out by the online version of the MedCalc software (MedCalc Software Ltd. Comparison of means calculator. https://www.medcalc.org/calc/comparison_of_means.php (Version 20.114; accessed September 4, 2022)). For the one-way analysis of variations test (ANOVA) a network applet was utilized (https://statpages.info/anova1sm.html). The applet also calculates the Tukey HSD (“Honestly Significant Difference”) *post-hoc* test, to indicate the significance between different groups. Unless otherwise stated, data shown represent means and standard deviations (±SD) from at least three independent biological determinations. The detailed data of the statistical analysis is included in the corresponding Supplementary information .xlsx files.

## Results

### Cell-cycle dependent analysis of γH2AX foci reveals attenuated repair of complex DSBs in G_2_-phase

Figure 1A shows the CHO clonal cell lines employed in the present study (described in detail earlier (Schipler et al., 2016)). For each clone, the type of integrated construct and the number of integrations measured by Southern blotting are indicated. The arrangement shows the simple forms of DSBs on top and the more complex forms at the bottom. Complexity is defined by the number of DSBs in the cluster and the type of apical ends generated assuming digestion of all I-*Sce*I sites. Thus, DSBs comprising compatible ends are considered simpler than those with incompatible ends. Owing to the integration of these constructs into the genome of CHO cells, DSBs are generated upon transfection of the I-*Sce*I expression vector, always at the same genomic locations. Note that after exposure to IR, DSBs are randomly generated throughout the genome. Therefore, in an irradiated cell population, no two cells will sustain DSBs at the same genomic locations. The fully defined nature of complexity and the precise induction of DSBs in the genome, both in terms of numbers as well as location, are the two key strengths of our model. A report published in 2016 provides evidence that despite different complexity, I-SceI expression induces one γH2AX focus per DSB cluster, independently of the number of DSBs it comprises (Schipler et al., 2016). Formation and repair of these DSBs obeys the specifics of I-SceI expression that starts a few hours after transfection and lasts 3 days or more (Schipler et al., 2016). The prolonged presence of I-SceI in transfected cells has as a consequence that repair events restoring the I-SceI recognition-site will allow re-cutting and will generate a “chronic” DSB. The first rejoining event that alters the I-SceI recognition site will be “terminal” and will mark, alternatively to I-*Sce*I extinction, the beginning of γH2AX focus-decay. These facts need to be considered in the analysis of γH2AX foci-formation and decay, as a proxy for the repair of the underlying DSBs.

**FIGURE 2:**
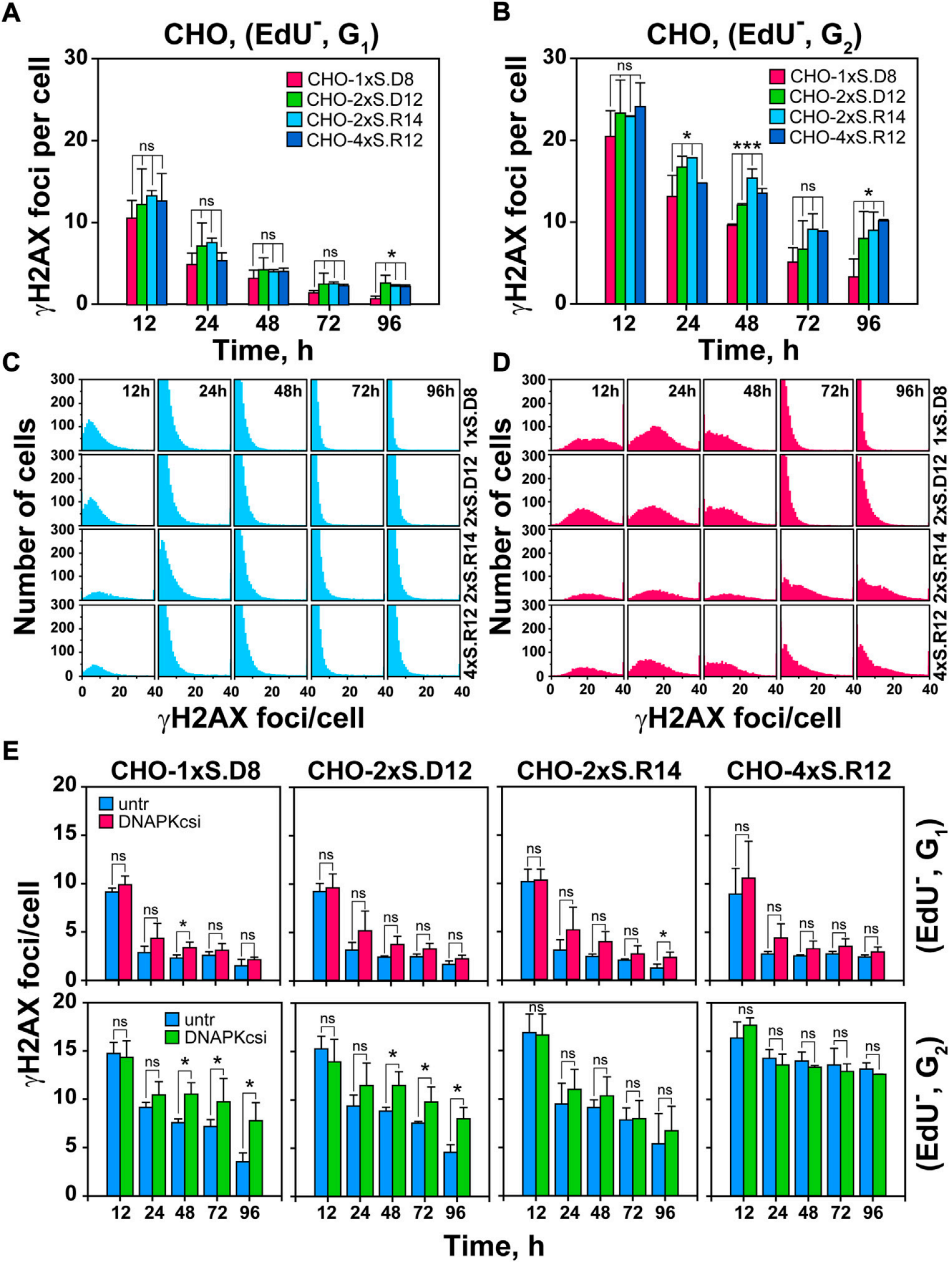
Increased DSB-complexity correlates with attenuated decay of γH2AX foci in G_2_-phase cells; impact of DNA-PKcs-inhibition. **(A)** Quantitative analysis (QA) of γH2AX foci formation and decay in G_1_-phase cells, selected according to the gates defined in Supplementary Figure S1A. Foci scored in I-SceI transfected cells is subtracted from the background foci scored in mock transfected cells. **(B)** As in Figure 2A, but for EdU^−^, G_2_-phase cells **(C)** Histogram plots of γH2AX foci in EdU^−^, G_1_-phase cells. **(D)** As in Figure 2C, but for G_2_-phase cells. **(E)** *Upper Panels:* QA of formation and decay of γH2AX foci in I-SceI transfected, G_1_-phase cells, after DNA-PKcs inhibition. DNA-PKcsi was administered immediately after transfection and was kept for 24 h. *Lower Panels:* As in the *upper panels* but for EdU^−^, G_2_-phase cells. Data represent means and standard deviations (±SD) from three independent experiments. (ns, no significance, *p* > 0.05); (*p ≤ 0.05); (**p ≤ 0.01); (***p ≤ 0.001); (****p ≤ 0.0001).

To facilitate the interpretation of the results obtained, we follow here γH2AX foci in a cell-cycle-dependent manner, for up to 96 h after I-SceI transfection. We focus on the G_1_-and G_2_-phase compartments, as delimited by DAPI-signal intensity in EdU^−^ cells (EdU given 30ʹ before processing of each time point), to reduce complications from background signals generated in S-phase cells (Figures 1B, 2 and Supplementary Figure S1A). An additional reason for this choice is that recent work demonstrates unexpected but highly significant mechanistic shifts in DDR and DSB-repair, depending not only upon the phase of the cell-cycle cells are irradiated, but also the phase of the cell-cycle responses are analyzed (Mladenov et al., 2019a; Mladenov et al., 2019b; Mladenov et al., 2020). We note however that because, in contrast to IR, damage induction by I-*Sce*I is not instantaneous but protracted, our cell-cycle-specific analysis is devoid of information on the phase of the cell-cycle DSBs are generated.

Figures 2A,B and Supplementary Figure S1C show that in both G_1_-and G_2_-phase, γH2AX foci-formation reaches a maximum at 12 h and declines thereafter. As expected from the doubling of DNA content, G_2_-phase cells display about twice the number of foci measured in G_1_–phase cells and appear to retain them for longer. There is a trend, more dominant in G_2_ – than in G_1_-phase cells, for γH2AX foci to decline faster in clones with “simple” DSBs than in clones with complex DSBs. This analysis becomes more informative when analyzing γH2AX foci distribution per cell (Figures 2C,D). Whereas in G_1_–phase, foci reduction occurs similarly at all levels of DSB-complexity, clearly more cells with large numbers of foci are scored in G_2_-phase for DSB-doublets and quadruplets with incompatible ends, i.e. for complex DSBs.

### Clustered DSBs suppress the engagement of c-NHEJ

To obtain information on the repair pathways processing DSBs of increasing complexity in different clones, we inhibited DNA-PKcs, the key kinase of c-NHEJ, with NU7441 and follow γH2AX foci in a cell-cycle-dependent manner, for up to 96 h after I-SceI transfection. Notably, this treatment has only a small effect on DSB-repair in G_1_–phase, mostly 24- and 48 h post-transfection, at all levels of complexity. However, in G_2_-phase cells, NU7441 only suppresses repair of single DSBs (Figure 2E), suggesting that, similarly to previous observations using different endpoints (Pang et al., 2011; Schipler et al., 2016), DSB-clusters fail to engage c-NHEJ. We conclude that in G_2_-phase, complex DSBs engage resection-dependent DSB-repair pathways that remain unaffected by DNA-PKcs inhibition.

### Increased contribution of HR to the processing of DSB-clusters

**FIGURE 3:**
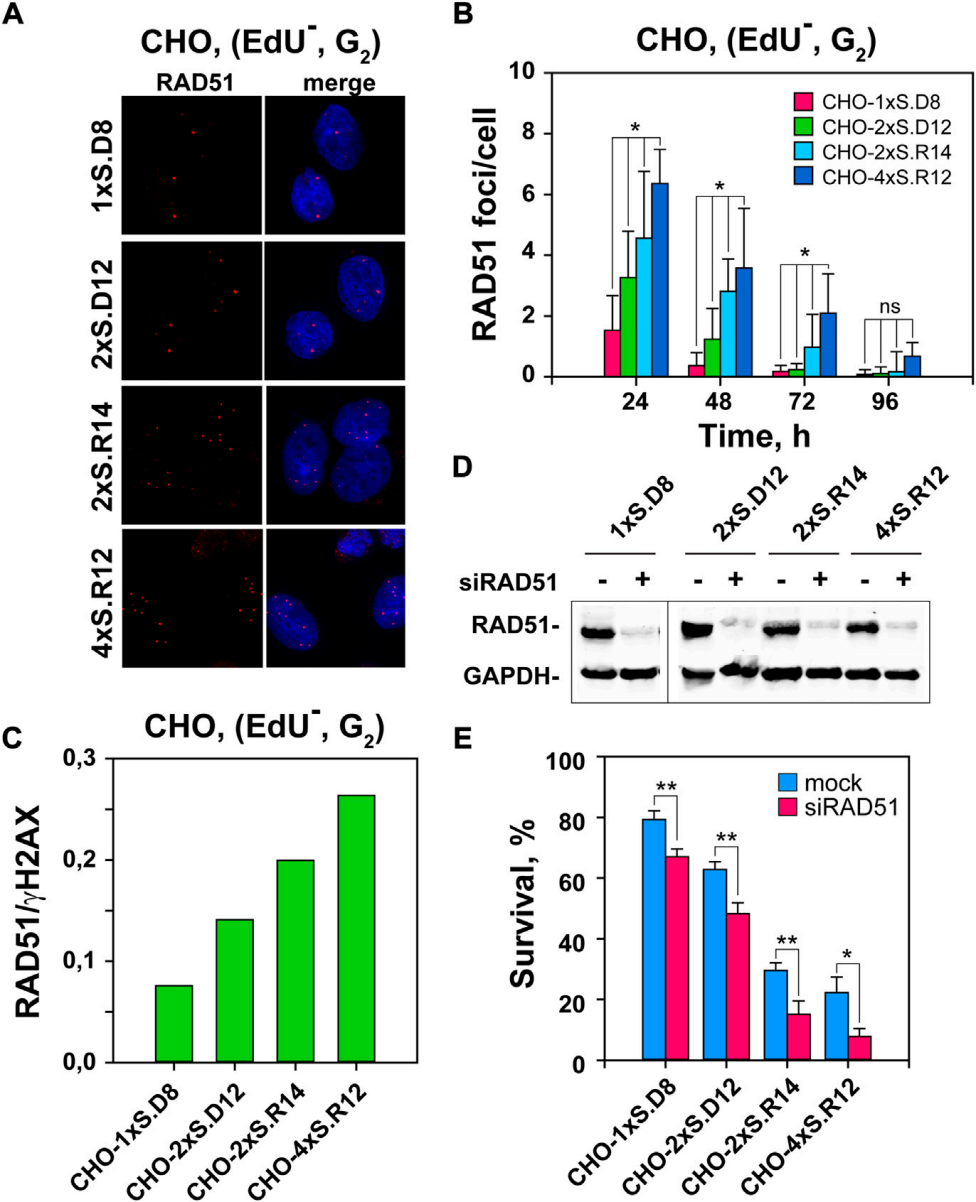
Stronger RAD51 accumulation to DSB-clusters than individual DSBs. **(A)** Representative images of RAD51 foci in I-SceI-transfected CHO clones. **(B)** QIBC of RAD51 foci, specifically in the EdU^–^G_2_-phase cells. The selection of EdU^–^, G_2_-phase cells is according to the gates defined in Supplementary Figure S1B. Only results for EdU–, G_2_-phase cells are presented, as there are no detectable RAD51 foci in the G_1_-cell population. **(C)** Ratio between RAD51 and γH2AX foci in the indicated CHO clones, as a measure of the fraction of DSBs that engage HR. It is calculated using the data from the 24 h time point, where the maximum in RAD51 foci is observed and γH2AX foci numbers are close to the maximum measured at 12 h **(D)** Western-blot analysis showing the level of RAD51 depletion. **(E)** Survival of transfected cells after depletion of RAD51 recombinase by siRNA interference calculated using the plating efficiency measured in mock-transfected cells of the corresponding clone. Data represent means and standard deviations (±SD) from three independent experiments. (ns, no significance, *p* > 0.05); (*p ≤ 0.05); (**p ≤ 0.01); (***p ≤ 0.001); (****p ≤ 0.0001).

To examine the contribution of HR to the repair of DSB-clusters, we scored RAD51 foci as a function of time after I-*Sce*I transfection, specifically in EdU^−^ G_2_-phase cells (Figure 3 and Supplementary Figure S1B). RAD51 accretion to DSBs is detectable at 8 h post transfection and reaches a maximum at 24 h (Figures 3A,B). Notably, QIBC results show that in cells, where complex DSBs are generated, RAD51 foci develop to a greater extent (Figure 3B). Indeed, nearly fourfold more RAD51 foci are scored at clusters of four DSBs, as compared to single DSBs. Calculation of the proportion of DSBs processed by HR by calculating the ratio of RAD51 to γH2AX foci shows, despite the above discussed caveats associated with these estimations, a clear increase with increasing DSB-complexity (Figure 3C). However, it is notable that even at the highest level of RAD51 foci measured in the clone with DSB-quadruplets, only ∼25% of DSBs are processed by HR – leaving 75% to be processed by other repair pathways. Since we show above that these DSBs also suppress the engagement of c-NHEJ, we infer that these remaining DSBs are repaired either by alt-EJ or SSA.

To substantiate the contribution of HR to the repair of complex DSBs, we studied the impact of RAD51 depletion by RNA interference on cell viability. Figure 3D indicates efficient knockdown with the selected siRNAs. Notably, RAD51-knockdown selectively sensitizes to killing cells that sustain complex DSBs (Figure 3E and Supplementary Figure S2A). We conclude that HR is preferentially involved in the processing of complex DSBs.

### DSB clusters increase the incidence of SCAs

**FIGURE 4:**
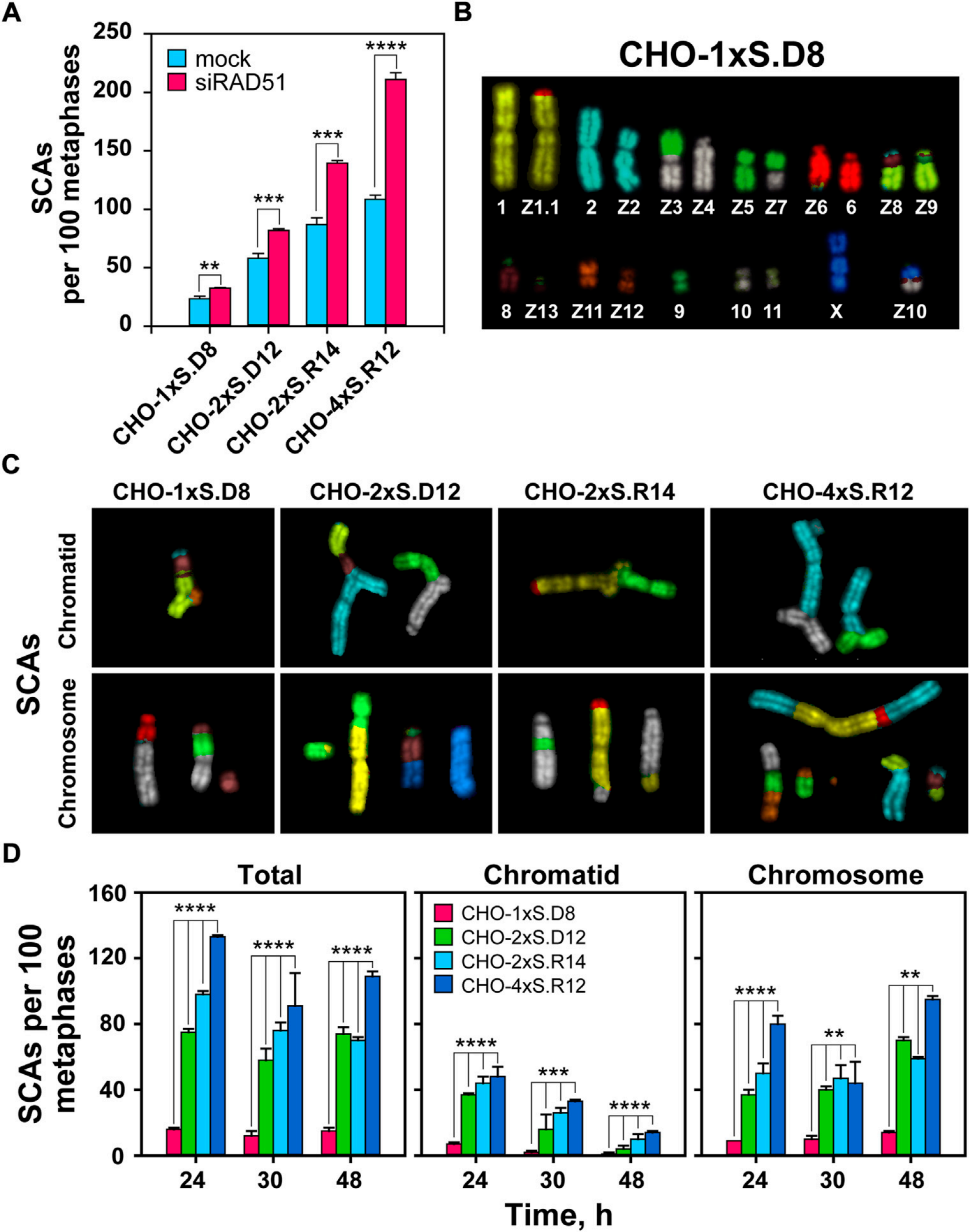
mFISH analysis of SCAs following induction of single and clustered DSBs. **(A)** QA of SCAs scored 24 h after I-SceI transfection in RAD51-depleted CHO clones. **(B)** mFISH karyotype of CHO-1xS.D8 cells. Note: the karyograms of undamaged chromosomes in all CHO clones are identical with the karyogram of CHO10B4 cells. **(C)** Representative images of chromatid- and chromosome-type SCAs captured 24 h after I-*Sce*I transfection. **(D)** QA of chromatid and chromosomal abnormalities scored at the indicated times after transfection of the indicated CHO clones. The numbers of SCAs scored in mock-transfected cells have been subtracted from the results presented. At least 100 metaphases are scored per metaphase spread. Data represent means and standard deviations (±SD) from three independent experiments. (ns, no significance, *p* > 0.05); (*p ≤ 0.05); (**p ≤ 0.01); (***p ≤ 0.001); (****p ≤ 0.0001).

А recent study shows that complex DSBs in the form of DSB clusters are markedly more efficient in generating SCAs than single DSBs (Schipler et al., 2016). It is also known that high-LET IR induces complex SCAs (Anderson et al., 2002), whose incidence correlates with the increased lethality observed. Here, we extend these studies using mFISH. For karyotyping, we follow the previously published chromosome annotation (Wurm and Hacker, 2011) of CHO cells (Figure 4B). Karyotyping of the parental CHO10B4 cell line and the derived clones shows no detectable changes among them. The analysis also reveals several stable, reciprocal translocations, as well as aneuploidy (2n = 21), inherent in this cell line (Figure 4B).

We assessed the potential of simple and clustered DSBs to form SCAs (Figures 4C,D). As a first step, we use classical cytogenetics and confirm our previous results by showing increased levels of SCAs with increasing DSB-complexity. Notably, we also observe that suppression of HR increases SCAs formation and conclude that HR suppresses not only killing, but also SCAs formation in cells sustaining complex DSBs (Figure 4A and Supplementary Figure S3).

mFISH data also confirm the above results (Figure 4D). As expected, the total number of SCAs is higher compared to those obtained using classical cytogenetics, which reflects the superior detection potential of mFISH analysis (Figure 4D). Interestingly, with increasing post-transfection time, we observe a shift in the ratio between chromatid- and chromosome-type aberrations (Figures 4C,D), indicating progression of cells through more than one cell-cycles and the conversion plus transmission of structural changes initially affecting chromatids, to chromosome type alterations (Huret et al., 2001).

**FIGURE 5:**
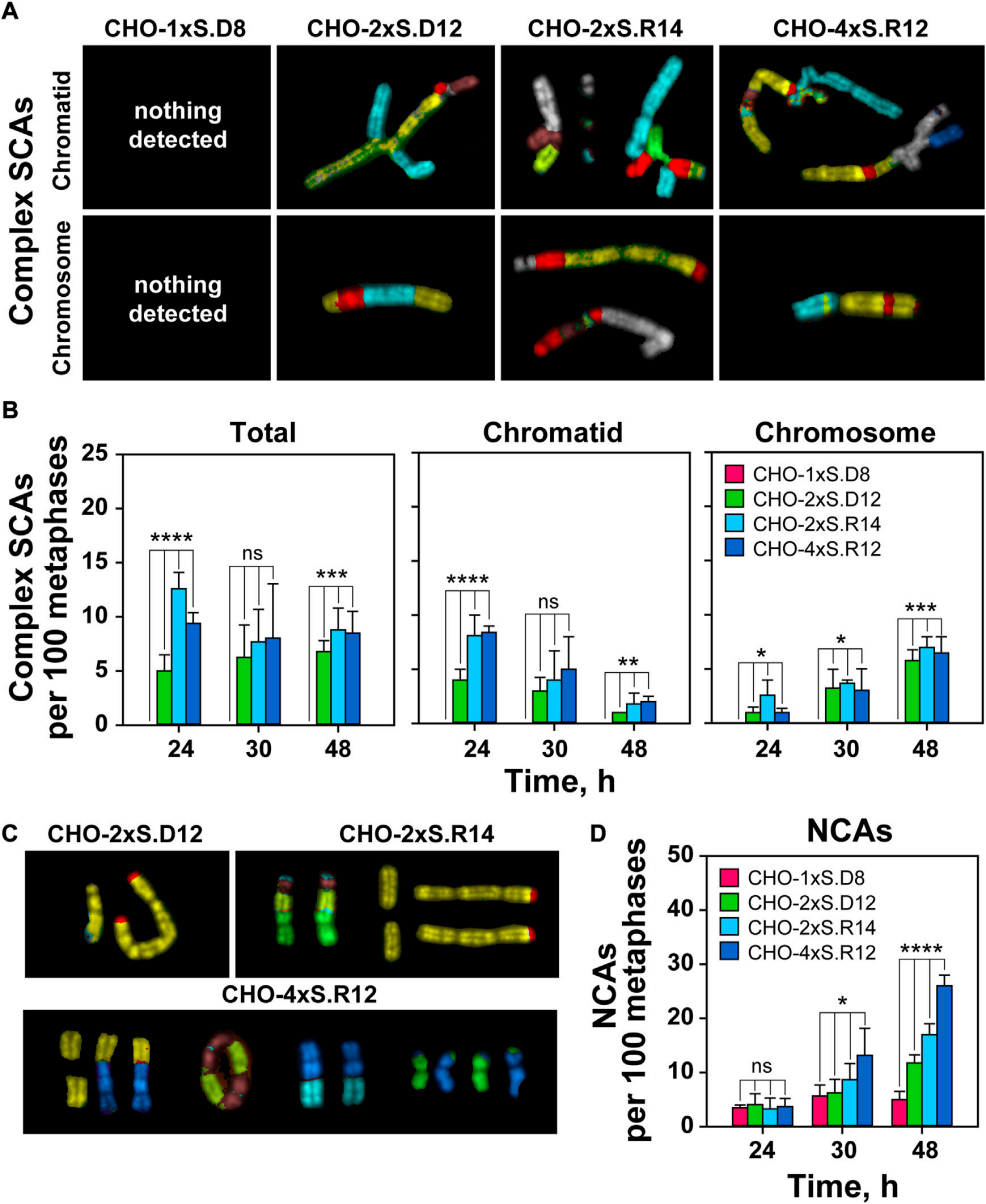
Increased DSB-complexity correlates with increased incidence of complex SCAs and NCAs. **(A)** As in Figure 4C but for complex SCAs. **(B)** As in Figure 4D but for complex SCAs. **(C)** Representative mFISH images of selected NCAs captured 48 h after transfection. **(D)** QA of NCAs. At least 100 metaphases are scored per sample. Data represent means and standard deviations (±SD) from three independent experiments. (ns, no significance, *p* > 0.05); (*p ≤ 0.05); (**p ≤ 0.01); (***p ≤ 0.001); (****p ≤ 0.0001).

Similar conclusions are drawn from the analysis of complex SCAs (Figure 5A). However, cells sustaining complex DSBs with incompatible ends show increased incidence of complex SCAs (Figure 5B). Notably, the incidence of complex SCAs in cells sustaining DSB-doublets with compatible ends remains low. Complex SCAs are not detected in cells sustaining individual DSBs (Figures 5A,B).

**FIGURE 6:**
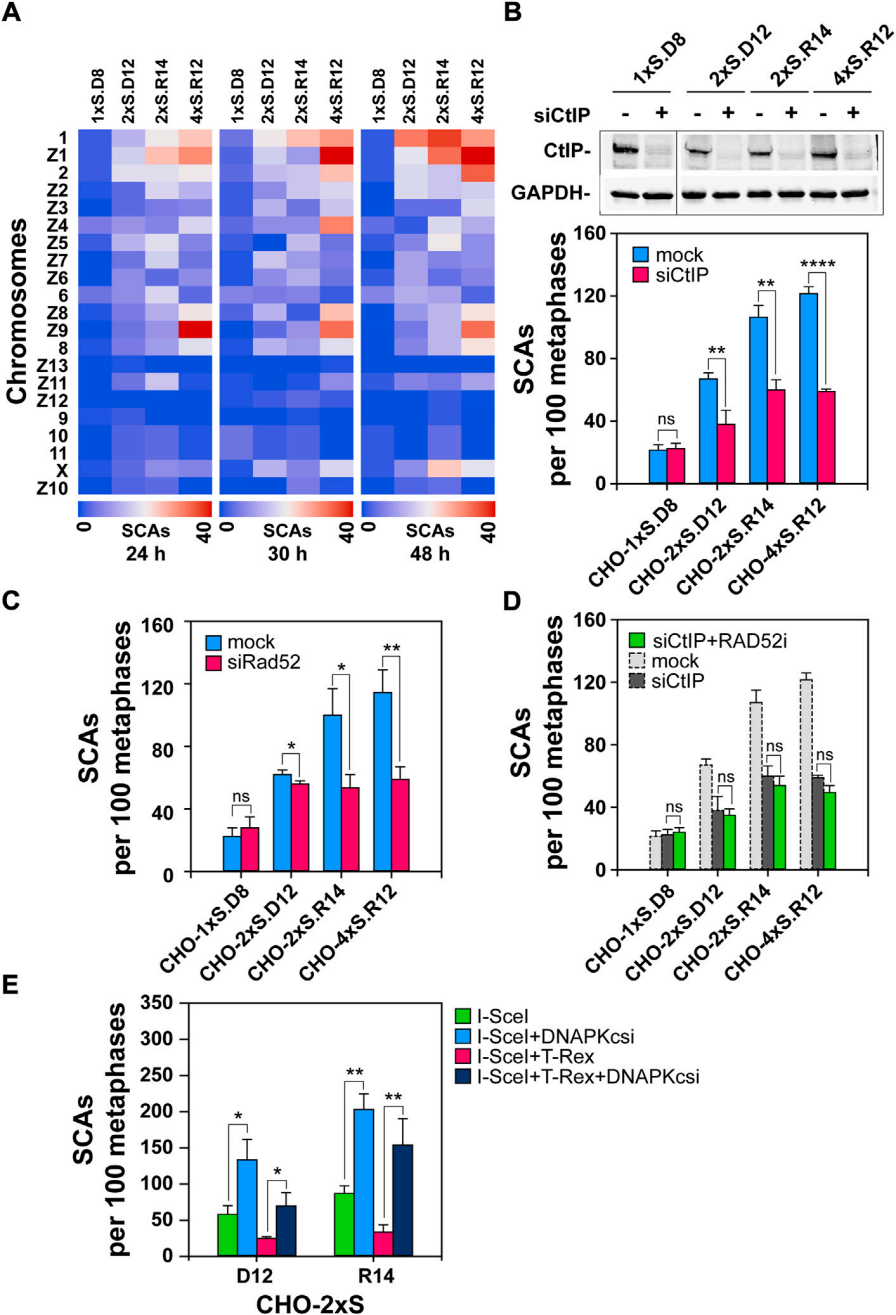
Depletion of CtIP or RAD52 and blunting of DNA-ends reduces SCAs in cells harboring DSB-clusters **(A)** Heat-map plots generated on the Orange software platform showing SCAs-frequencies of individual hamster chromosomes in the indicated clones. **(B)** *Upper Panel:* Western-blot analysis showing CtIP knock-down in the indicated clones; *Lower Panel:* QA of SCAs scored 24 h post-I-SceI transfection following CtIP depletion. **(C)** As in Figure 6B, *Lower Panel,* but for RAD52-depleted cells **(D)** QA of SCAs scored 24 h post I-*Sce*I transfection following CtIP depletion, combined with RAD52 inhibition. RAD52i was administered immediately after transfection and was kept for 24 h. Results represent means and SD from at least three independent experiments; only two independent experiments are conducted for CtIP-depletion in combination with RAD52 inhibition. **(E)** SCAs scored 24 h post transfection with either the I-SceI expression vector alone, or together with the TREX-expression vector in CHO-2xS.D12 or CHO-2xS.R14 cells, in the presence or absence of DNA-PKcsi. At least 100 metaphases are scored per sample. Data represent means and standard deviations (±SD) from three independent experiments. (ns, no significance, *p* > 0.05); (*p ≤ 0.05); (**p ≤ 0.01); (***p ≤ 0.001); (****p ≤ 0.0001).

Since I-*Sce*I DSBs are not randomly distributed, but are localized at the sites of construct integration in the CHO genome, we assessed the frequencies of SCAs at each individual chromosome. Our results suggest that some chromosomes preferentially sustain SCAs – from the non-random integration of I-*Sce*I constructs into the clone-genome (Figure 6A). The heat-map-plots indicate that there are at least two groups of chromosomes (the first group includes chromosomes 1, Z1 and 2 and the second chromosomes Z8, Z9 and 8), which more frequently participate in SCAs; this trend is preserved and further enhanced 48 h after transfection. The heat-maps also reveal that the frequency of SCAs at the X-chromosome increases at later time points, indicating another SCAs hot-spot and possibly also a different mechanism of formation. We are presently sequencing the genome of our clones to determine the precise locations of the I-*Sce*I-construct integrations.

We also observe a correlation between DSB-cluster formation and increased frequency of numerical chromosome abnormalities (NCAs) (Figures 5C,D). The number of such events increases with time after transfection. There is a strong increase in NCAs at 48 h post-transfection in clones with complex DSB-clusters, whereas clones harboring individual I-SceI sites show less time-dependent increase in NCAs. Overall, the results show that increased DSB-clustering results in extensive deletions and driftage apart of diverse acentric chromosome fragments (AFs) (Supplementary Figure S2B). Such AFs, or even whole chromosomes, could be potentially incorporated into micronuclei that are considered genotoxic events and signs of chromosomal instability (Durante and Formenti, 2018).

### Abrogation of resection suppresses SCAs-formation at complex DSBs

A recent study shows that inhibition of alt-EJ using PARP1 inhibitors abrogates SCAs-formation, and that this effect is stronger for DSB-clusters (Schipler et al., 2016). Here we complement these studies by investigating the role of resection in SCAs formation, which is an operational requirement for all DSB-repair pathways, except c-NHEJ. Resection is initiated by the MRN/CtIP complex and therefore we started by knocking-down CtIP. Western-blot analysis 24 h post-transfection shows efficient knock-down of the protein in all CHO clones (Figure 6B). Notably, CtIP knockdown has no, or only a marginal, effect on SCAs-formation in cells sustaining single DSBs or pairs with compatible DNA-ends. On the other hand, CtIP knockdown markedly inhibits SCAs-formation in clones sustaining complex DSB with incompatible ends (Figure 6B).

To assess the contribution of SSA to SCAs-formation under these conditions, we depleted RAD52, 24 h before transfection for I-SceI expression. Similar to CtIP depletion, RAD52 knockdown has no impact on SCAs in cells sustaining individual DSBs and has no statistically significant effect on cells sustaining DSB pairs with compatible ends (Figure 6C). However, upon RAD52 knockdown, a significant reduction in the number of SCAs is observed in cells sustaining complex DSBs with incompatible ends (Figure 6C). Expectedly, inhibition of RAD52 in cells depleted for CtIP fails to further enhance SCAs-formation (Figure 6D), confirming the postulated contribution of SSA.

### TREX processing of DNA-ends suppresses SCAs-formation

To evaluate whether processing of the DSB-overhangs present at I-*Sce*I induced DSBs (Figure 1A) modulates SCAs-formation, we adopted an approach in which DSB-ends generated by I-SceI in CHO-2xS.D12 and CHO-2xS.R14 are modified by transient expression of TREX, fused to I-SceI. TREX is a non-processive 3′-exonuclease that can degrade the 3′-overhangs generated by I-SceI (Certo et al., 2012). This processing generates blunt DNA-ends and may modulate SCAs-formation. To investigate this possibility, we co-transfected cells with pCMV-3xNLS-I*Sce*I and pI*Sce*I-T2A-Trex2-IRES-BFP and scored SCAs 24 h later. Notably, a reduction by 60–70% in SCAs-formation is noted in both CHO-2xS.D12 and CHO-2xS.R14 cells upon I-SceI and TREX co-expression (Figure 6E). This result suggests that single-stranded overhangs at the DNA-ends facilitate the processes underpinning SCAs-formation. Furthermore, our results also show that blunted DNA-ends are processed by DNA-PKcs, and therefore DNA-PKcsi causes a nearly six-fold increase in SCAs in the CHO-4xS.R12 clone (Figure 6E).

**FIGURE 7:**
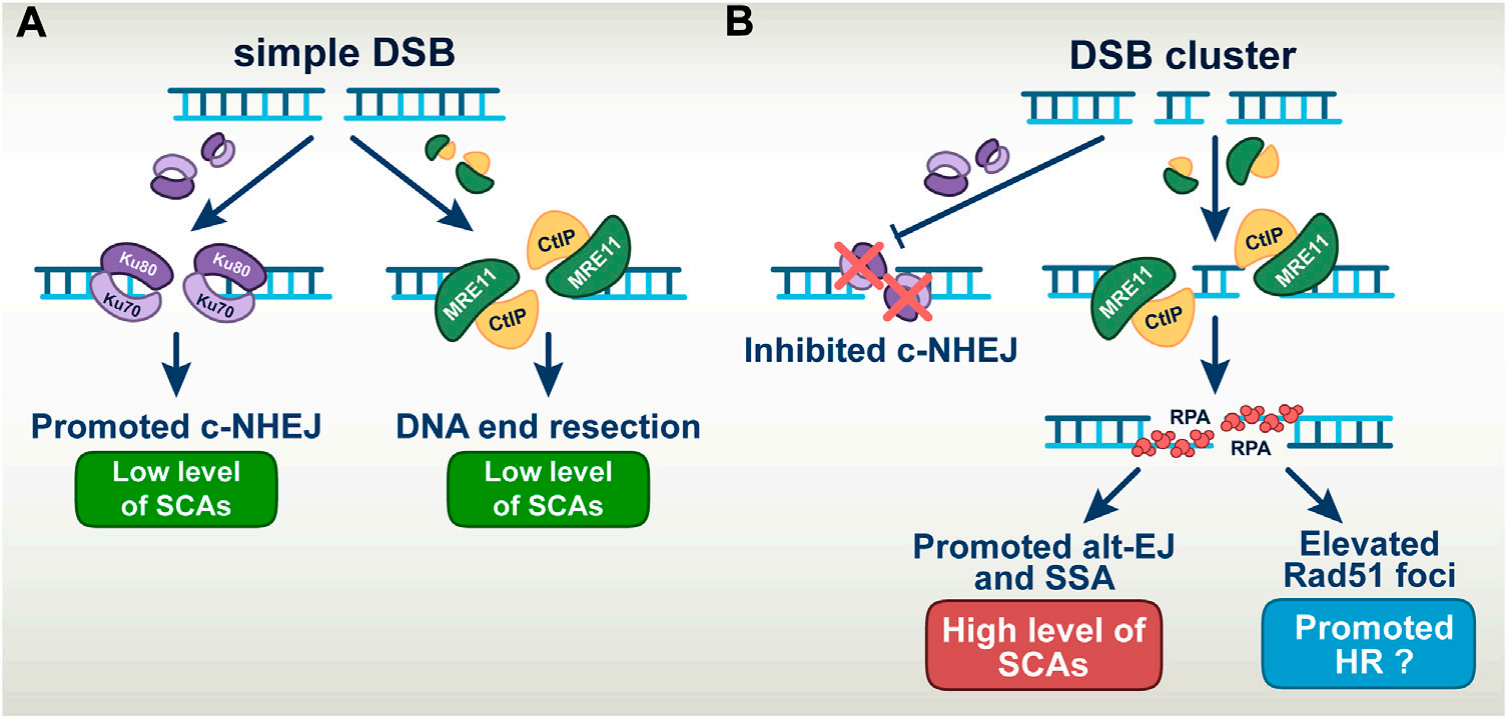
A model describing the altered DSB repair pathway balance in simple *versus* complex DSBs **(A)** Simple DSBs engage repair by both c-NHEJ and DNA end-resection dependent pathways, which results in low level of SCAs formation. **(B)** DSBs clusters suppress the repair by c-NHEJ thus allowing the involvement of error-prone DNA end-resection dependent repair pathways. This manifests in the increased level of SCAs when alt-EJ and SSA are involved in repair. Elevated number of RAD51 foci indicates the involvement of HR in the repair of DSB-clusters; However, it remains unclear to what extent HR is productive.

In aggregate, our results confirm previous findings that DSB-clusters suppress c-NHEJ, through mechanisms, which still remain poorly understood. We conclude that the high toxicity of DSB-clusters modelling high-LET-DNA damage derives from promotion of alt-EJ and SSA despite increases in HR. (Figure 7).

## Discussion

The results summarized above show that increased DSB-complexity, as defined by I-*Sce*I-induced DSB-clustering and the ligatability of the generated apical DNA-ends, has profound implications to DSB-processing and that this processing exhibits marked cell-cycle dependencies. Thus, while DSB-repair (γH2AX-foci-decay) shows only relatively small changes with increasing DSB-complexity in cells analyzed in G_1_-phase, it causes pronounced delays in cells analyzed in G_2_-phase. Furthermore, the observation that processing of complex DSBs in G_2_-phase is resistant to DNA-PKcs inhibitors, not only explains this delay, but also suggests that DSB-complexity is a strong suppressor of c-NHEJ (Schipler et al., 2016).

A key characteristic of G_2_-phase cells with reference to DSB-processing that also distinguishes them from G_1_ -phase cells, is that resection and HR are fully active. We therefore infer that resection and HR are intimately contributing to the observed distinct outcomes in G_2_-phase cells. Indeed, we show that HR engagement increases with increasing DSB-complexity (Figure 3). This result is in line with reports of increased HR-engagement in cells exposed to high-LET IR and validates the simulation potential of our model for high-LET IR-effects. This similarity of response further suggests that DSB clusters are a highly consequential form of complex DNA damage induced by high-LET IR.

Notably, despite increased engagement of HR on complex DSBs, more than 75% of them are still processed by other repair pathways. Since c-NHEJ fails to engage on complex DSBs, it follows that the remaining 75% are processed by alt-EJ or SSA. Since these pathways are inherently error-prone, the results allow us to resolve the conundrum as to why and how increased engagement of HR in high-LET irradiated cells fails to make them radioresistant. Evidently, the majority of complex DSBs are repaired under these conditions by highly error-prone repair pathways leading to the enhanced cell killing and genomic instability observed. The choice among these pathways may be regulated by the degree of resection, an aspect that is presently under investigation (Sallmyr and Tomkinson, 2018; Seol et al., 2018; Jalan et al., 2019). These aspects of DSB-processing on simple and complex DSBs are summarized in a graphical manner in Figure 7. Notably, the enhanced role of HR generates opportunities for further radiosensitization of cells exposed in high-LET IR, a point we return to below. It is also highly relevant that the suppression of c-NHEJ causes a switch to resection-dependent DSB-processing at complex DSBs and places the process of resection at the forefront.

Since error-prone DSB-processing causes SCAs, we analyzed the mechanisms underpinning their formation as a function of DSB-complexity. Indeed, the increases in DSB-complexity modelled here (Figure 1A), lead to remarkable increases in SCAs-formation (both simple and complex, and extending to NCAs) (Figures 4, 5). Marked increases in SCAs-formation are also observed after exposure to high-LET IR, further validating our model system (Franken et al., 2012; Loucas et al., 2013). However, when cells are exposed to high-LET IR the presence of a wide spectrum of additional lesions randomly distributed in the genome seriously limits the interpretation of the results obtained and their mechanistic analysis.

Notably, suppression of HR increases SCAs-formation and sensitizes cells to complex DSBs, as expected from an error-free repair pathway promoting genomic stability and cell survival (Figure 4A and Supplementary Figure S3). Ablation of resection by CtIP knockdown has a small effect on SCAs-formation from simple DSBs, but generates a profound suppression of SCAs from complex DSBs. This implies that SCAs-formation from complex DSBs requires resection. It is important to point out that suppression of resection will also strongly suppress HR, and as a consequence estimates of “real” suppression should be made by taking the increased SCAs-levels measured following RAD51 knockdown into consideration.

Knockdown of RAD52, a key component of SSA, strongly suppresses SCAs-formation (Figure 6C) confirming thus the involvement of SSA in their formation. It has been previously reported that inhibition of PARP1, a component of alt-EJ, also strongly suppresses SCAs-formation (Wray et al., 2013; Schipler et al., 2016). Collectively, these results provide thus conclusive evidence for a dominant role of alt-EJ and SSA in high-LET genomic instability and define targets for enhancing their effects, by additional suppression of error-free DSB-repair pathways like HR.

It is intriguing that SCAs-formation utilizes the 4 bp TTAT 3′-overhangs generated by I-*Sce*I, as their removal by TREX strongly suppresses SCAs-formation. It is equally interesting that DNA-ends apparently blunted by TREX, can be processed by DNA-PKcs even when highly complex, as inhibition of DNA-PKcs dramatically increases SCAs-formation. The result of this inhibition further indicates that blunted DNA-ends, actually good substrates of DNA-PKcs (Lieber, 2010), can be effortlessly shunted to alt-EJ or SSA, upon DNA-PKcs inhibition, to effectively form SCAs. This result points to repair pathway choice dynamics that are intimately regulated by relatively small modifications at the DNA-ends and which have hitherto remained elusive – pointing again to the power of our model system.

The important role of resection in the processing of modelled complex DSBs in G_2_-phase has important parallels to the effects of high-LET IR as compared to low-LET IR. Indeed, the enhanced resection initiated at complex DSBs predicts increased engagement of ATR as it is observed (Xue et al., 2015; Mladenov et al., 2019a; Mladenov et al., 2019b; Mladenova et al., 2021). There are reports showing that ATR is recruited and activated at DSBs after exposure of cells to high-LET IR, and that under these conditions ATR also regulates resection. The same holds true for the activation of the G_2_-checkpoint (Xue et al., 2015; Mladenov et al., 2019a; Mladenov et al., 2019b; Mladenova et al., 2021). The high-LET IR-dependent stimulation of resection is also supported by the 53BP1 and RPA chromatin dynamics after exposure to X-rays and α-particles (Roobol et al., 2020). Interestingly, extended cell-cycle analysis demonstrates that resection-promoting factors are present at DSBs generated by high-LET IR, even in G_1_-phase, where resection is normally suppressed (Averbeck et al., 2014).

The expansion of particle therapy using protons or HI offers new opportunities for improving cancer care (Durante et al., 2017; Durante, 2019), but requires profound understanding of molecular mechanisms underlying the higher effectiveness of high-LET IR. We provide here for the first time some important advances in this regard that offer concrete strategies to improve these forms of therapy. A model system generating DSBs of molecularly defined complexity has been instrumental in this analysis. Our results provide strong rationale for the use of HR inhibitors, as well as ATRi (which are also known to indirectly suppress HR) in particle therapy to further sensitize tumor cells and also suggest the use of RAD52 and PARP1 inhibitors to further enhance efficacy. Modulation of the engagement of high-fidelity HR and highly error prone alt-EJ and SSA has also direct potential in radiation protection of astronauts during space travel.

## Data Availability

The raw data supporting the conclusions of this article will be made available by the authors, without undue reservation.

## Abbreviations

AFs, Acentric chromosome fragments; HI, Heavy ions; NCAs, Numerical chromosomal abnormalities; QA, Quantitative analysis; QIBC, Quantitative image-based cytometry; SCAs, Structural chromosomal abnormalities.
