# Supplementary material for: The high toxicity of DSB-clusters modelling high-LET-DNA damage derives from inhibition of c-NHEJ and promotion of alt-EJ and SSA despite increases in HR
Source: Front Cell Dev Biol. 2022 Oct 3;10:1016951. doi: 10.3389/fcell.2022.1016951 (PMC9574094; doi:10.3389/fcell.2022.1016951)
Supplement: Supplementary file 4 [file DataSheet1.PDF]

Supplementary Material

1. Supplementary Figures

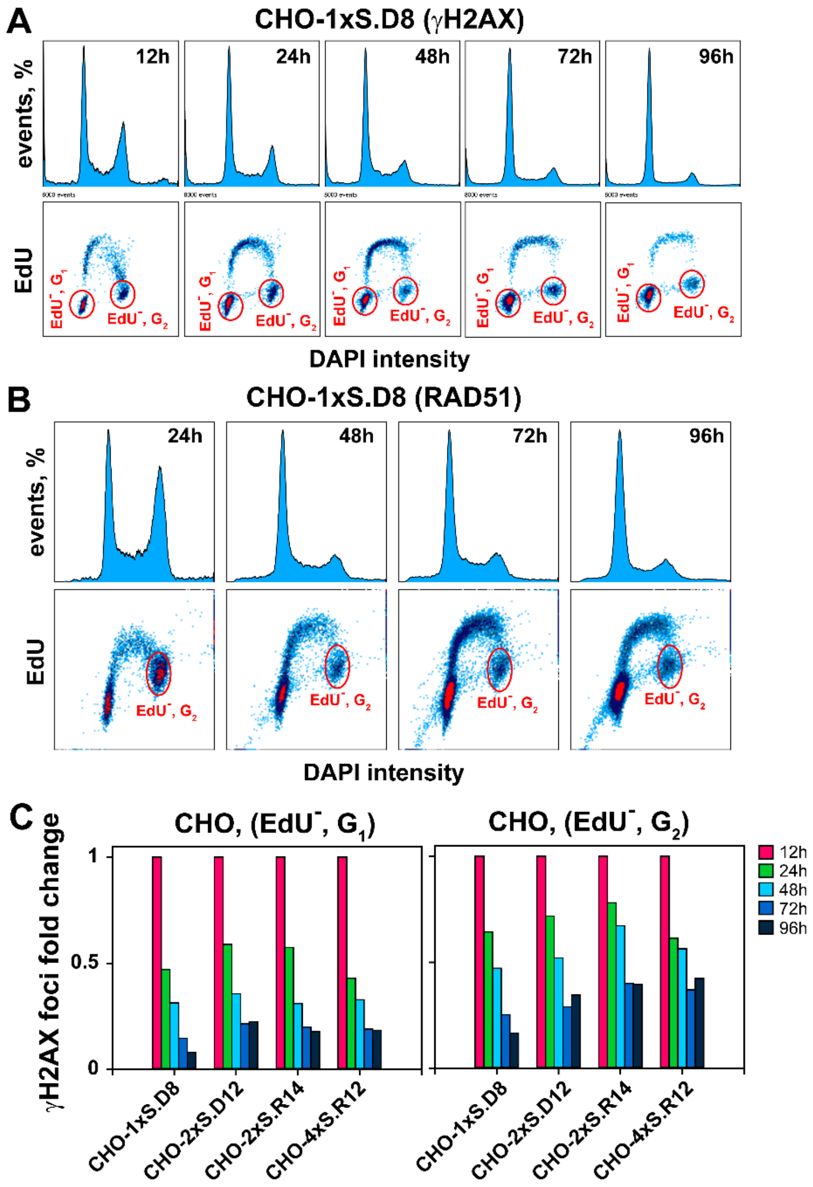

Supplementary Figure 1. (A) Upper Panels: Histogram plots showing the distribution of cells in the cell-cycle determined by DAPI intensity measurements during γH2AX QIBC-analysis; Lower Panels: scattered plots of EdU vs. DAPI signal in the QIBC-analysis of the same cells. The gates used to select G_1- (EdU^-, G_1) and G_2-phase cells (EdU^-, G_2) are shown. (B) As in **Suppl. Figure 1A**, but for QIBC-analysis of RAD51 foci. (C) Fold change of γH2AX foci number determined in Figure 2.

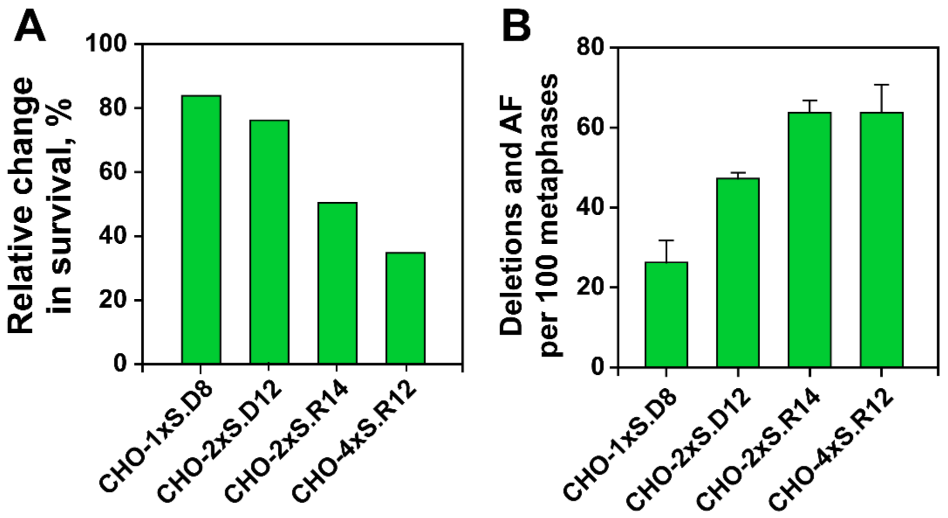

Supplementary Figure 2. (A) Relative changes in survival of the indicated CHO clones following RAD51 depletion and I-SceI expression. Values are normalized to survival values of controls transfected for I-SceI expression only. (B) QA of larger deletions and shorter acentric fragments (AFs) scored at metaphase 24h after I-SceI transfection of the indicated CHO clones. The numbers of deletions and AFs scored in mock-transfected cells have been subtracted from the results shown.

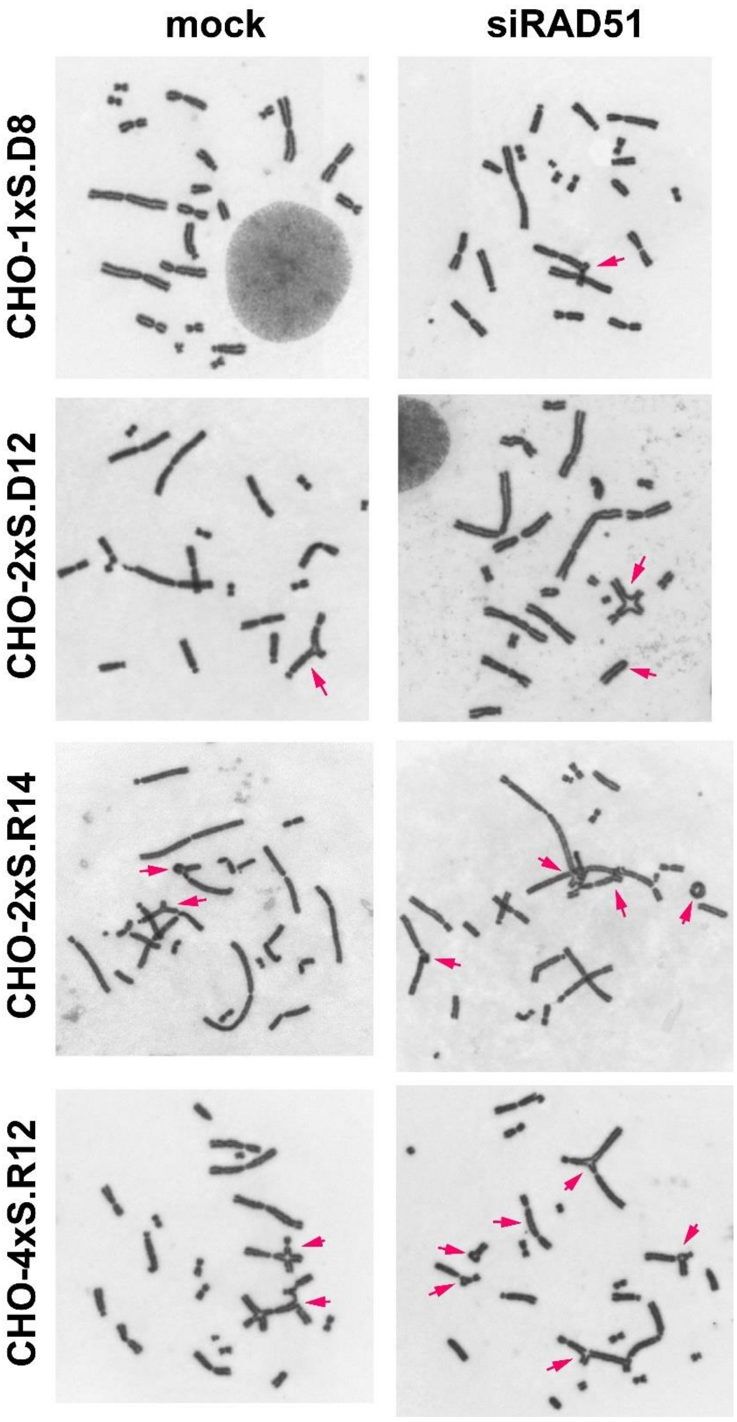

Supplementary Figure 3. Representative images of SCAs generated in the indicated clonal cell lines following downregulation of RAD51 and transient transfection for I-SceI expression. SCAs were scored 24h after transfection; metaphase chromosome spreads were prepared and fixed using standard cytogenetic procedures.
